# Medical Entomology

**Published:** 1910-05-28

**Authors:** 


### Medical Entomology.

The Secretary of State for the Colonies has appointed an "Entomological Research Committee (Tropical Africa)" for the purpose of organising and co-ordinating the investigation of the numerous insects which have from time immemorial been the bane of the great Dark Continent. The Chairman of this Committee is Lord Cromer, who continues to lay both Africa and his own country under the greatest obligations to himself after services such as perhaps no other living Englishman has already rendered. His colleagues include many well-known scientists, and already their energy has resulted in the publication of an important periodical devoted entirely to the furtherance of medical entomology. The first number of this *Bulletin of Entomological Research* has been forwarded to these offices, and it is no exaggeration to say that it promises to aid the forces of civilisation and progress in Africa to a most marked degree. Dr. A. E. Shipley contributes a preface, in which he outlines the need for the work which this Committee is to set in hand, the nature of the field to be reaped, and the plans projected for the development of the enterprise. As an economic problem the subject should possess the greatest possible fascination for anyone who is interested in the study of the rise and fall of nations and empires; for it is not too much to say that the cause of the almost complete closure of Africa until recent times (with the exception of the very narrow fringe along the Mediterranean and the lower Nile) has been the existence of the ticks and flies which carry death and destruction to man and beast. Dr. Shipley also directs attention to the other side of the picture when he says: "On the other hand, it must not be forgotten that many insects are beneficial to man. More than one kind of ladybird . . . introduced from Australia, has done much to free the Californian orange trees from that destructive pest, the fluted scale . . . and Dr. Sharp states that if anything were to exterminate the enemies of Hemiptera, we ourselves should probably be starved in the course of a few months. If possible, it is important to find out what insects are helpful to man in any new country before the advent of large numbers of colonists upsets, as it is bound to do, the balance of power in the animal world."

The services which Dr. Shipley has rendered to entomology in general, and to tropical entomology more particularly, are many and various. None of them, we can safely assert, has been more useful than the way in which he has induced the Colonial Office to take up this question. It is pleasing, therefore, to notice in the public Press that an attempt is being made to recognise in some way these services by raising a special fund for the purpose of presenting this eminent zoologist with his portrait, to be painted by Mr. William Nicholson, on behalf of the many friends he has made in all quarters of the globe, and last, but by no means least, on behalf of the men he has trained to take a practical and useful interest in entomological research.

Arrangements have been made through the Colonial Office by which the Committee will be kept in touch with the organisations that already exist, both to the north with the authorities of the Soudan and Egypt, and to the south with Rhodesia and the United South African States. The need for such an organisation is apparent when Dr. Shipley's statements on the arithmetic of entomology are digested. In 1830 the number of insects described and named, and in most cases figured, was under 50,000; by 1881 it had risen to over 220,000. Since the latter date fully as many more new species have been discovered, so that nearly 450,000 are now on the list. Thousands more await classification and description in collections deposited with the important museums where no one has time to work them out, and it is calculated those yet uncaptured run into millions. In promoting the work of sorting out and studying the life cycles of this vast horde of creatures the Committee has decided to send out two well-known and experienced entomologists, one to East Africa and one to West Africa; these gentlemen have already left England, bound, respectively, first of all for Nyassaland and Nigeria. Their duties will include the instruction of residents, especially Government servants, in the methods of collecting and studying ticks and insects and of preserving them for transmission home. According to present knowledge, many large orders, such as the Lepidoptera and Neuroptera, exert but little influence on human affairs; and, in view of the enormous task that lies before the Committee, it is probably a wise decision to confine attention for the present to such insects and ticks as bite or sting man and his domesticated animals with their mouth-parts, or are parasites of man and his animals, and to those which may be responsible in any other way for the transmission of disease or are destructive to crops and timber. In course of time the vermian and protozoal parasites must also be investigated; but this is being deferred as less urgent than the other investigation.

The field it will be seen is a very vast one, and there are at present comparatively few labourers in it, although it is satisfactory to note that, mainly owing to the active interest taken by the Colonial Office in the matter, several young entomologists are now engaged in the investigation of special insect pests in various parts of the Crown Colonies. The Dominions possess their own independent Government entomologists, who have done excellent work, both special and general, in the past, and may be confidently expected to do as good work in the future.

In the furtherance of this comprehensive programme the publication of the *Bulletin* is bound to exercise a most helpful influence. In the first number the most important article is one by Mr. W. Wesché on the larval and pupal stages of West African Culicidæ. The greater part of this long and valuable research is necessarily technical, but as an introduction the author provides it with some most instructive and practical hints on the main principles of examination, couched in language which is simple yet accurate, and should enable even those with but little previous experience to identify their captures and determine their new discoveries. The *Bulletin* is excellently printed and mounted, as, indeed, what is virtually a Government publication should be; altogether it and the similar publication of the Sleeping Sickness Bureau are two efforts on behalf of suffering humanity far more likely to yield really great results than anything—the abolition of the slave trade excepted—that Great Britain has yet attempted in Africa.

